# Romiplostim-related myelofibrosis in refractory primary immune thrombocytopenia: A Case report

**DOI:** 10.1097/MD.0000000000015882

**Published:** 2019-06-21

**Authors:** Hyun-Young Kim, Sung Woo Park, Jung Hoon Kim, Jung Hun Kang, Won Seop Lee, Haa-Na Song

**Affiliations:** a Department of Laboratory Medicine, Gyeongsang National University of Medicine and Gyeongsang National University Hospital; b Division of Hemato-oncology, Department of Internal Medicine, Gyeongsang National University of Medicine and Gyeongsang National University Hospital, Republic of Korea.

**Keywords:** myelofibrosis, romiplostim, thrombocytopenia

## Abstract

**Rationale:**

Primary immune thrombocytopenia (ITP) is an immune-mediated disease that is defined as increased platelet destruction and impaired platelet production. Treatment is recommended for highly selected patients, the standard regimen includes glucocorticoid, intravenous immunoglobulin (IVIG). The recombinant thrombopoietin (TPO) receptor agonists, romiplostim, stimulate platelet production and have approved for glucocorticoid or IVIG, splenectomy-refractory chronic ITP patients.

**Patient concerns:**

A patient has been diagnosed with ITP, reftractory to steroid, IVIG, splenectomy, danazol, and cyclosporine. The patient received romiplostim to normalize his platelet count, however, over the course of the following year, his platelet counts progressively decreased despite increasing the romiplostim dosing.

**Diagnoses:**

A peripheral blood smear showed a severe leukoerythroblastic reaction and bone marrow biopsy demonstrated myelofibrosis due to romiplostim.

**Outcomes:**

Since this diagnosis, romiplostim was discontinued for a while, after 3 months, romiplostim was re-administered to improve thrombocytopenia. His platelet count recovered to 70,000/mm^3^ after the administration of romiplostim at 2 μg/kg, and he did not experience complications for 6 months.

**Lessons:**

This report represents the first evidence of romiplostim-induced myelofibrosis, which was associated with increased levels of bone marrow reticulin and Masson trichrome staining.

## 1 Introduction

Primary immune thrombocytopenia (ITP) is an immune-mediated disease that is defined by increased platelet destruction and impaired platelet production, which result in increased bleeding risk in the absence of an underlying cause.^[1]^ The primary treatment is based on the severity of the bleeding symptoms and platelet counts. For patients with platelet counts above 30,000/mm^3^, unless accompanied by other bleeding risks that include trauma and surgery, mandated anticoagulation therapy is not necessary. ^[2]^ Treatment is recommended for patients with platelet counts lower than 10,000/mm^3^, regardless of bleeding signs, and for patients with platelet counts between 10,000 and 30,000/mm^3^ and significant bleeding symptoms. First-line treatment for ITP includes glucocorticoids, intravenous anti-(Rh)D, and intravenous immunoglobulins (IVIG).^[3]^ Second-line treatments include splenectomy, azathioprine, cyclosporine A, cyclophosphamide, danazol, dapsone, and rituximab.^[4]^ Recombinant thrombopoietin (TPO) receptor agonists, romiplostim and eltrombopag, stimulate platelet production and have been approved for the treatment of patients with chronic ITP who showed poor responses after glucocorticoid, IVIG, or splenectomy interventions. ^[5]^

In this report, we present the case of a 53-year-old man with chronic ITP who was refractory to standard treatment, received romiplostim, and presented secondary myelofibrosis. The Institutional Review Board of Gyeongsang National University of Hospital approved this retrospective case study and waived the requirement for informed consent.

## 2 Case report

A 53-year-old man with chronic ITP presented with petechiae in both upper extremities for 3 days. He was diagnosed with ITP eight years ago; however, a high-dose steroid and immunoglobulin treatment failed. After the failed treatment, he underwent a splenectomy and received cyclophosphamide for 2 years. However, he experienced a thrombocytopenia recurrence and was prescribed danazol for 2 years, after which he received cyclosporine for refractory ITP. His platelet count was poorly controlled by cyclosporine, so eltrombopag, an oral TPO-agonist, was prescribed. Since he did not take his medication regularly, his platelet count did not return to the normal range. After self-discontinuation of eltrombopag, he was lost to follow-up.

**Figure 1:**
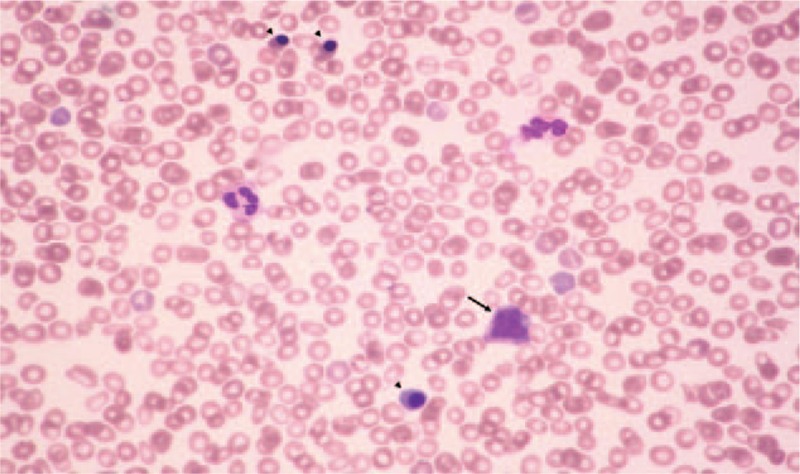
A peripheral blood smear (×400) showed nucleated RBCs (arrow head) and myelocytes (arrow).

**Figure 2:**
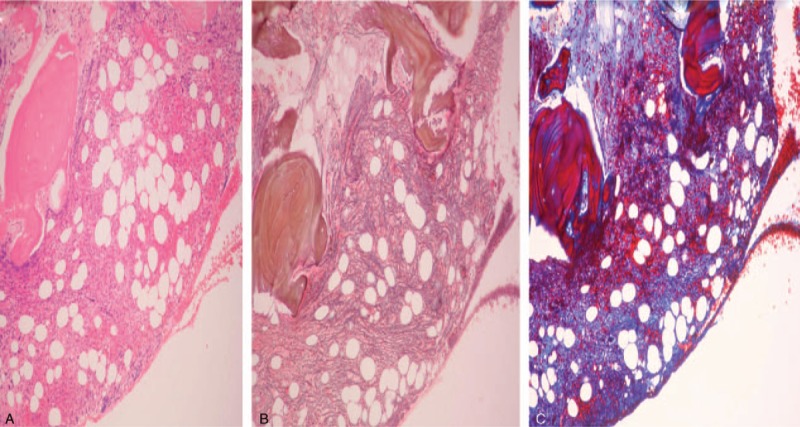
A: Bone marrow biopsy showed hypercellular marrow with increased megakaryocytes and myelofibrosis (H&E stain, ×100). B: A reticulin stain and C: Masson trichrome stain revealed an increase in diffuse and dense reticulin fibers with focal collagen bundles (MF-2) (×100).

About one year later, he was admitted to our hospital for multiple petechiae in his extremities, and his platelet count was 7000/mm^3^. He received romiplostim, a subcutaneously injected thrombopoietin (TPO) agonist, and his platelet count recovered to 65,000/mm^3^. After discharge, he visited our hospital for weekly romiplostim injections. Initially, he responded to the treatment, and his platelet counts increased to 80,000 to 100,000/mm^3^. However, over the course of the following year, his platelet counts progressively decreased, despite an increased romiplostim dose, to 9 μg/kg per week. During this period, he also developed anemia, due to decreased hemoglobin levels (9 g/dL). A peripheral blood smear showed a severe leucoerythroblastic reaction (Fig. 1); however, the bone marrow aspiration smear was inadequate for evaluation, and the bone marrow biopsy demonstrated hypercellular marrow, with 90% cellularity, proliferation of pleomorphic megakaryocytes, and myelofibrosis (Fig. 2A). A reticulin stain and Masson trichrome stain revealed an increase in diffuse and dense reticulin fibers, with focal bundles of collagen (MF-2) (Fig. 2B, C). The patient was diagnosed with romiplostim-induced myelofibrosis. Since this diagnosis, romiplostim was discontinued, and the patient received platelet apheresis transfusions every three months.

Along with his worsening thrombocytopenia, the patient developed dyspnea and chest discomfort. He was referred to the cardiology department for an evaluation of his symptoms, and an echocardiography was performed, which showed normal results. However, the coronary angiography showed significant LAD (left anterior descending coronary artery) stenosis, and a percutaneous coronary intervention with a LAD stent insertion was performed. Due to his antiplatelet agent prescription, romiplostim was re-administered to improve thrombocytopenia. His platelet count recovered to 70,000/mm^3^ after the administration of romiplostim at 2 μg/kg, and he did not experience complications including myelofibrosis for 6 months.

## 3 Discussion

Romiplostim, a TPO receptor agonist, is used to control thrombocytopenia by stimulating megakaryocyte proliferation and differentiation; it also increases platelet response to agonists. Megakaryocyte proliferation and differentiation are activated via the Janus-type tyrosine kinase (JAK) – signal transducer and activator of transcription (STAT) and mitogen-activated protein (MAP) kinase pathways. Weekly subcutaneous romiplostim injections at doses that start at 1 μg/kg produce a dose-dependent increase in platelet counts. The romiplostim dose that was used for this patient was increased by 1 μg/kg, to a maximum of 10 μg/kg, until his platelet count reached 50,000/mm^3^.

Previous studies have reported that the platelet responses were achieved in about 80% to 90% of patients with ITP who were treated with romiplostim. ^[6]^ The common adverse effects (AEs) were fatigue, headache, epistaxis, thrombosis, and infection. ^[7]^ Especially, Kuter et al. showed that bone marrow reticulin increased after 7 weeks of romiplostim treatment; however, the reticulin level returned to baseline 14 weeks after romiplostim discontinuation.^[8]^ More advanced studies revealed that about 1% to 4% of patients with chronic ITP who received romiplostim had increased bone marrow reticulin levels, and this effect was usually reversible and dose dependent. Eleven of 292 patients with ITP showed increased reticulin, and one patient was diagnosed with secondary myelofibrosis that was due to reticulin, not trichrome. ^[9]^ Pulikkan et al investigated the bone marrow fibrosis pathway by modulating the thrombopoietin (THPO) pathway, via activating the MPL/PI3K/AKT axis. In a mouse model, higher fibrosis was observed after TPO transfection. ^[10]^

This study has several limitations. First, after re-administration of romiplostim, we did not perform re-biopsy of bone marrow to confirm romiplostim-induced myelofibrosis. Instead, we estimated from the results of peripheral blood count, including anemia and thrombocytopenia, was improved after re-administration of romiplostim. Also, like many other case series, this study shows lack of evidence due to its retrospective design and danger of over-interpretation.

We present the case of a patient with chronic ITP who experienced secondary myelofibrosis and received romiplostim. The bone marrow biopsy shown increased reticulin levels, and the Masson trichrome stain and the peripheral blood smear showed severe leukoerythroblastosis. To the best of our knowledge, this report represents the first evidence of romiplostim-induced myelofibrosis, which was associated with increased both levels of bone marrow reticulin and Masson trichrome staining. In summary, romiplostim-induced myelofibrosis should be considered in patients with chronic ITP, despite treatment effectiveness.

## Author contributions

**Conceptualization**: Sung Woo Park, Haa-Na Song.

**Data curation**: Jung Hoon Kim

**Formal analysis**: Haa-Na Song

**Investigation**: Jung Hun Kang, Won Seop Lee

**Resources**: Haa-Na Song

**Writing – original draft**: Hyun-Young Kim

**Writing – review and editing**: Haa-Na Song

Haa-Na Song orcid: 0000-0002-7229-0153
